# Outcomes and Follow-Up Data From Two Skin Cancer Screening Events

**DOI:** 10.7759/cureus.43938

**Published:** 2023-08-22

**Authors:** Jade N Young, Nithya Krishnamurthy, Annie Chang, Dina Poplausky, Nicholas Gulati, Jonathan Ungar

**Affiliations:** 1 Dermatology, Icahn School of Medicine at Mount Sinai, New York, USA

**Keywords:** skin screening outcomes, dermatology screening, free skin screening, skin cancer screening, skin cancer

## Abstract

This study investigated the outcomes and follow-up behaviors of participants from two free skin cancer screening events in the United States. This survey, with 296 participants and a 31% response rate, gathered information on participant demographics, personal history of skin cancer, knowledge of skin screening practices, and follow-up behaviors. There was a high follow-up rate of 92.3% among individuals recommended for further dermatological consultation, but a low (22%) concordance rate between the preliminary diagnoses from the screening and patient-recalled diagnoses. Additionally, about one-sixth of participants identified limited access to care as a motivation for participating in the screening. The study emphasizes the need to improve awareness about the limitations of free screenings, enhance participant education, and ensure equitable access to skin cancer screening. Future research should focus on factors influencing follow-up behaviors and the development of targeted interventions to increase awareness and access to skin cancer screening.

## Introduction

Skin cancer represents the most prevalent form of cancer in the United States, however, there is a paucity of data surrounding the effectiveness of routine skin screenings within the low-risk population [1]. This may stem from variability among skin cancer screening programs and an insufficiency of quality improvement research in the literature. To promote sun-protective behaviors and increase access to dermatologic care, many dermatologists conduct free screening events for the general community [2,3]. These free screenings are similar to clinical skin cancer screenings but do not involve diagnostic procedures or treatments. Numerous studies have examined the rates of skin cancer diagnosis and the number needed to screen to detect one skin cancer during screening events [4-8] but only a handful of studies have examined follow-up behaviors of individuals participating in free screenings when faced with lesions that necessitate further evaluation [7].

Additional research has shed light on the need for nationwide melanoma screening programs, as highlighted by Curiel-Lewandrowski et al [9]. Implementing such programs can lead to improved early detection of skin cancer and a reduction in the incidence of thicker melanomas. Primary care providers play an important role in skin cancer screening and prevention, and Oliveria et al. found that strategies to increase skin cancer prevention by family practitioners need to be considered [10]. Thus, increased education regarding sun protection and skin cancer should be utilized in this model [11,12]. These prevention efforts through skin cancer screening may help to curb the rising incidence of skin cancer. 

While the benefits of free skin screenings have been demonstrated, participant comprehension and diagnosis recollection of free skin screenings have yet to be assessed. Outcome analysis and follow-up data are crucial components of enhancing the effectiveness of skin cancer screening initiatives. Particularly, it is important that participants understand the limitations of free screening events and have a clear understanding of the next steps should action be required. To bridge the existing gaps, this study sought to characterize the outcomes and assess follow-up data procured from two skin cancer screening events through a post-screening survey. Ultimately, this study aims to contribute to improving skin cancer screening initiatives and early detection efforts.

## Materials and methods

This study was a cross-sectional assessment of free skin screening participants who attended two skin cancer screening events: one took place in Aspen, Colorado, in June 2022, and the other in New York, New York, in May 2022. A 28-question survey was distributed to participants through REDCap software (Vanderbilt University, Nashville, Tennessee) six to seven months after the screenings. The survey was distributed to a total of 296 participants who provided contact information for follow-up. The response rate was expected to be reduced as a consequence of the latency between survey administration and event attendance, however, a follow-up period of at least six months was chosen to ensure the capture of proper follow-up. The survey aimed to gather information on participant demographics (race, ethnicity, income, education), personal history of skin cancer, family history of skin cancer, knowledge of skin screening practices, identification of any flagged lesions during the screening, and subsequent post-screening follow-up behaviors including biopsy results. 

The statistical analysis was conducted using R version 4.2.1 (R Foundation for Statistical Computing, Vienna, Austria). Descriptive statistics were employed to evaluate demographic characteristics. Concordance between the self-reported preliminary diagnoses and physician-recorded preliminary diagnoses was assessed. The concordance rate and follow-up rate were reported as percentages. Participants were categorized into two groups based on their access to healthcare services: those with limited access, defined as individuals who reported difficulty in scheduling appointments with a dermatologist, lack of access to a dermatologist, or absence of health insurance, and those with unrestricted access. The correlation between access to care and dermatologic outcomes was assessed using chi-square and Fisher's exact tests.

## Results

The survey was sent to 296 individuals and received 93 responses (31% response rate). The full survey results are displayed in Tables 1-4. Around 99% of participants indicated they would recommend the screening to family and friends. A majority (80%) of participants had undergone a previous skin screening with a dermatologist. Only 41% of participants recognized that there is a difference between a free screening event and a screening in a dermatologist’s office. A significant association was found between having prior access to care and a prior history of skin cancer (p = 0.006). Among the 23 individuals recommended for a follow-up appointment with a dermatologist, a high follow-up rate of 91.3% (21/23) was observed. The percentage of individuals recommended for a follow-up in the whole cohort (28%) was equivalent to that of the survey responders (28%). Regarding insurance status, no significant association with follow-up rates was observed. Self-reported preliminary diagnoses of participants who followed up with a recommended dermatologist appointment included basal cell carcinoma (3/23), squamous cell carcinoma (2/23), melanoma (1/23), and actinic keratosis (4/23). Of the remaining lesions, 8/23 indicated “I don’t know” and 3/23 indicated “other.” Diagnostic concordance data on the concordance between these self-reported preliminary diagnoses and those documented on the screening form by the physician was assessed. Among the 23 lesions that were flagged as suspicious at the screening, only 22% (5/23) of patients reported a preliminary diagnosis concordant with the one given at the time of screening. There were 14/23 lesions for which self-reported biopsy data were available. Self-reported biopsy-proven diagnoses included basal cell carcinoma (3), squamous cell carcinoma (2), actinic keratosis (1), and benign lesions (6). Two participants reported not knowing their final biopsy results. Of the nine participants that did not provide biopsy data, 3/9 were presumed actinic keratoses.

**Table 1:** Demographics Demographic information of participants

Demographic characteristics of the participants	
Race/Ethnicity	
White	79% (73/92)
Hispanic/Latino	9% (8/92)
Asian	3% (3/92)
Black	3% (3/92)
American Indian	1% (1/92)
Other	4% (4/92)
Income	
$0-25K	5% (4/88)
$25-75K	8% (7/88)
$75-150K	19% (17/88)
$150-250K	23% (20/88)
>$250K	45% (40/88)
Education	
High school	1% (1/93)
Bachelor’s degree	26% (24/93)
Master’s degree	46% (43/93)
Doctorate	24% (22/93)
Associate degree	2% (2/93)
Insurance	
Private Insurance	74% (69/93)
Medicare	20% (19/93)
Medicaid	2% (2/93)
No insurance	1% (1/93)
Other	1% (1/93)

**Table 2:** Skin Cancer History Personal and family skin cancer history among participants

Medical History	Past Medical History	Past Family History
Basal Cell Carcinoma	19% (18/93)	20% (19/93)
Squamous Cell Carcinoma	7% (7/93)	17% (16/93)
Melanoma	2% (2/93)	25% (23/93)
Other	3% (3/93)	4% (4/93)
Don't Know	11% (10/93)	22% (20/93)
None of the Above	66% (61/93)	32% (30/93)

**Table 3:** Survey Responses Survey questions and responses

Question	Yes	No	About the Same
Have you ever had a skin screening by a dermatologist in a doctor's office (i.e. not at a screening event)?	80% (74/93)	20% (19/93)	n/a
Have you ever had a skin screening by any health care provider (excluding a dermatologist) in a doctor's office (i.e. not at a screening event)?	43% (40/92)	57% (52/92)	n/a
Do you wear sunscreen regularly?	80% (74/93)	20% (19/93)	n/a
Is there a difference between the free skin screening you had and a skin screening at a dermatologist's office?	42% (38/90)	58% (52/90)	n/a
During the free skin screening, did the dermatologist recommend you make a follow-up appointment with your doctor or dermatologist?	28% (23/89)	72% (66/89)	n/a
After attending the free skin screening, would you recommend a skin screening to your family and friends?	99% (92/93)	1% (1/93)	n/a
After attending the free skin screening, do you feel more confident in self-examinations?	41% (38/93)	16% (15/93)	43% (40/93)
After attending the free skin screening, did you learn about sun protection?	64% (59/92)	36% (33/92)	n/a

**Table 4:** Skin Screening Motivations and Attitudes Survey responses detailing participant motivations for participating in the skin screening. Participants were permitted to choose more than one response. Attitudes regarding the free skin screening experience were also surveyed and recorded.

Why did you decide to participate in the free skin screening?	Counts
I had spots on my skin that were concerning to me	48% (45/93)
I wanted to learn more about skin	30% (28/93)
No reason, I was just walking by	19% (18/93)
I have had difficulty getting an appointment with my dermatologist	16% (15/93)
I don't have a dermatologist	15% (14/93)
I don't have health insurance	1% (1/93)
Other	8% (7/93)

## Discussion

The results of this study offer insights into the demographic characteristics, screening behaviors, and outcomes of two free skin cancer screening programs. A majority (99%) of participants indicated they would recommend the screening to others, suggesting a positive perception of the programs’ value and potential benefits such as the discovery of unknown skin cancers. This emphasizes the need for further promotion and expansion of such initiatives to increase awareness and educate individuals about skin cancer and sun protection [3,11,12]. There was a notable lack of confidence in self-examinations among participants following the screening and only a moderate increase in sun protection knowledge. These areas represent potential targets for improvement in education and counseling during future free skin screenings. These modifications could be easily streamlined into the skin screening programs through pamphlets or informational videos in the waiting room. 

The predominant motivations for attending the screening regarded specific concerns (“I had spots that were concerning to me”) and educational purposes (“I wanted to learn more about skin”). These motivations provide guidance for strategies to promote skin cancer screening events to the public centered around participants’ anticipated benefits. Approximately one-sixth of the responses cited limited access to dermatologic care as a motivation for attendance. The significant association between prior access to care and a history of skin cancer may indicate a higher rate of diagnosis among participants with access to dermatologists, rather than a true increased incidence. Our findings highlight how free screening events have the capability to engage individuals who may not typically have access to dermatology clinics [2]. Dermatologists should seek to host these events in rural and underserved areas to target this demographic who often lack skin cancer screening [13]. It should be emphasized, however, that the coordination of proper follow-up with dermatology or a primary care provider if necessary should be prioritized at events in these areas due to the potential lack of local providers. 

Most participants did not recognize the difference between a free skin cancer screening and a screening at the dermatologist’s office. Although free screenings provide an accessible option for skin cancer detection, they may not fully replace the expertise and comprehensiveness of a dermatologist's examination. This emphasizes the need for clear communication with patients regarding the limitations of free screenings, including lack of biopsy capability, and the importance of seeking further evaluation when necessary [1].

The high rate of follow-up within six months of the screening suggests that, of the participants who recalled needing follow-up, most successfully followed through with a dermatology appointment in a timely fashion. Insurance status did not affect the follow-up rate. In total, 14/23 patients received a biopsy as recommended, indicating a high rate of recommended follow-up and demonstrating the utility of free skin screenings in diagnosing malignant lesions. In addition, it is plausible that the nine participants who did not undergo a biopsy were not ultimately recommended to have one at their dermatology visit. For example, 3/9 with no biopsy were presumed actinic keratoses, which would not necessitate a biopsy. 

Among the lesions that were flagged as suspicious during the screening, a relatively low percentage were concordant with the diagnosis recalled by the patient during the survey. This sheds light on the importance of proper counseling and timely follow-up with dermatologists to ensure accurate diagnosis and appropriate treatment [7]. A potential solution could involve distributing fact sheets to the participants during the screening with information about the preliminary diagnosis, urgency, and next steps. 

Survey studies are inevitably limited by response bias. The participant cohort represents mostly Caucasian individuals of high socioeconomic status. There were very few uninsured participants or participants with Medicaid, introducing potential limitations in generalizability. In order to draw more generalizable conclusions, further studies should be conducted in regions of greater socioeconomic and ethnic diversity. Moreover, our study is limited by response rate. Future research should seek to elucidate the factors that influence screening attendance to develop targeted interventions that improve awareness of presumed diagnoses and access to skin cancer screening [14]. 

## Conclusions

Overall, the findings of this study underscore the need for ongoing efforts to improve awareness about the limitations of free screenings, including the inability to conduct diagnostic tests, and to ensure equitable access to quality care for all individuals. Further research is needed to explore the factors that influence follow-up behaviors and develop targeted interventions to improve participants’ awareness of presumed diagnoses and access to skin cancer screening. 
